# Engineering Targeting Materials for Therapeutic Cancer Vaccines

**DOI:** 10.3389/fbioe.2020.00019

**Published:** 2020-02-11

**Authors:** Priscilla S. Briquez, Sylvie Hauert, Alexandre de Titta, Laura T. Gray, Aaron T. Alpar, Melody A. Swartz, Jeffrey A. Hubbell

**Affiliations:** ^1^Pritzker School of Molecular Engineering, The University of Chicago, Chicago, IL, United States; ^2^Agap2 Zürich, Zurich, Switzerland; ^3^Ben May Department of Cancer Research, The University of Chicago, Chicago, IL, United States; ^4^Committee on Immunology, The University of Chicago, Chicago, IL, United States

**Keywords:** cancer, vaccines, material engineering, targeting strategies, immunoengineering, immunotherapy

## Abstract

Therapeutic cancer vaccines constitute a valuable tool to educate the immune system to fight tumors and prevent cancer relapse. Nevertheless, the number of cancer vaccines in the clinic remains very limited to date, highlighting the need for further technology development. Recently, cancer vaccines have been improved by the use of materials, which can strongly enhance their intrinsic properties and biodistribution profile. Moreover, vaccine efficacy and safety can be substantially modulated through selection of the site at which they are delivered, which fosters the engineering of materials capable of targeting cancer vaccines to specific relevant sites, such as within the tumor or within lymphoid organs, to further optimize their immunotherapeutic effects. In this review, we aim to give the reader an overview of principles and current strategies to engineer therapeutic cancer vaccines, with a particular focus on the use of site-specific targeting materials. We will first recall the goal of therapeutic cancer vaccination and the type of immune responses sought upon vaccination, before detailing key components of cancer vaccines. We will then present how materials can be engineered to enhance the vaccine’s pharmacokinetic and pharmacodynamic properties. Finally, we will discuss the rationale for site-specific targeting of cancer vaccines and provide examples of current targeting technologies.

## Introduction

Cancer ranks as the second leading cause of global deaths, according to the World Health Organization, with nearly 15% of people dying from it (World Health Organization, 2018). More alarmingly, the rate of cancer incidence is increasing and is expected to reach more than 20 million newly diagnosed cases per year and 13 million cancer-related deaths in 2030 (American Cancer Society, 2018; World Health Organization, 2018). Among the various types of cancer, the most prevalent are lung, liver, colorectal, stomach and breast cancers. While cancer can affect any part of the body and is very heterogeneous between patients, most malignant tumors share biological similarities – defined as the “hallmarks of cancer” (Hanahan and Weinberg, 2011) – which help researchers break down the disease complexity and subsequently guide them toward the development of effective cancer therapies.

Currently, surgery, radiotherapy and chemotherapy remain the first-lines of cancer treatments that are prescribed as a single therapy or in combination, in a patient-tailored fashion that depends on the tumor characteristics (e.g., type, stage, aggressiveness and accessibility), as well as the patient’s symptoms and health conditions (National Institutes of Health of the USA, 2019). In recent years, immunotherapies have emerged as highly promising treatments to educate the patient’s immune system to efficiently fight its own cancer cells. The most successful clinical cancer immunotherapies that have received United States FDA approval to date include adoptive T cells therapies, immunomodulatory therapies, targeted cancer therapies, oncolytic virus therapies and cancer vaccines, as detailed in Table 1. Some of these treatments are currently being established as first-line treatments in cases of advanced cancers (Peters et al., 2019), highlighting the strong clinical potential of such immunotherapies. Furthermore, a multitude of novel immunotherapeutic compounds are currently being tested in clinical trials, foreseeing a fast evolution of the cancer therapy landscape in upcoming years.

**TABLE 1:** Current approved United States FDA immunotherapies.

Type of immunotherapy	Immunotherapy	Drugs	Cancer types
Adoptive cell therapy	CD19-targeting CAR T cells	Tisangenlecleucel (Kymriah) Axicabtagene ciloleucel (Yescarta)	Leukemia, lymphoma, pediatric cancer
Oncolytic virus therapy	Herpes simplex virus	T-VEC (Imlygic)	Melanoma
Cancer vaccine	Bacillus Calmette-Guérin (BCG) vaccine *(therapeutic)*	BCG vaccine	Bladder cancer
	Human papilloma virus (HPV) vaccine *(preventive)*	Cervarix, Gardasil, Gardasil-9	Cervical cancer
	Hepatitis B virus vaccine *(preventive)*	Heplisav-B	Liver cancer
	Patient immune cells stimulated with PAP (prostatic acid phosphatase) (*therapeutic*)	Sipuleucel-T (Provenge)	Prostate cancer
Immunomodulator	Anti-PD-1/PDL-1	Atezolizumab (Tecentriq) Avelumab (Bavencio) Cemiplimab (Libtayo) Durvalumab (Imfinzi) Nivolumab (Opdivo) Pembrolizumab (Keytruda)	Bladder cancer, breast cancer, cervical cancer, Colorectal cancer, esophageal cancer, head and neck cancer, kidney cancer, liver cancer, lung cancer, lymphoma, melanoma, pediatric cancer, skin cancer, stomach cancer
	Anti-CTLA-4	Ipilimumab (Yervoy)	Melanoma, pediatric cancer
	Combination anti-PD-1 and anti-CTLA-4	Nivolumab (Opdivo) + Ipilimumab (Yervoy)	Kidney cancer, melanoma
	IL-2	Aldesleukin (Proleukin)	Kidney cancer, melanoma
	Interferon alfa-2a	Roferin-A	Leukemia
	Interferon alfa-2b	Intron A, Sylatron/PEG-Intron	Leukemia, lymphoma, melanoma
Other targeted therapies	Anti-GD2	Dinutuximab (Unituxin)	Brain cancer, pediatric cancer
	Anti-VEGF-R	Bevacizumab (Avastin) Ramucirumab (Cyramza)	Brain cancer, cervical cancer, colorectal cancer, esophageal cancer, kidney cancer, lung cancer, ovarian cancer, stomach cancer
	Anti-HER2 Anti-HER2-drug conjugates	Pertuzumab (Perjeta) Tratuzumab (Herceptin) Tratuzumab emtansine (Kadcyla)	Esophageal cancer, breast cancer, stomach cancer
	Anti-EGFR	Cetuximab (Erbitux) Necitumumab (Protrazza)	Colorectal cancer, head and neck cancer, lung cancer
	CD19-CD3 bispecific antibody	Blinatumomab (Blincyto)	Leukemia
	Anti-CD20 Anti-CD20-drug conjugates	Obinutuzumab (Gazyva), Ofatumumab (Arzerra), Rituximab (Rituxan) Ibritumomab tiuxetan (Zevalin)	Leukemia, lymphoma
	Anti-CD22-drug conjugates Anti-CD33-drug conjugates	Inotuzumab ozogamicin (Besponsa) Gemtuzumab ozogamicin (MyloTarg)	Leukemia
	Anti-CD52	Alemtuzumab (Campath)	Leukemia
	Anti-CD30-drug conjugates Anti-CD79b-drug conjugates	Brentuximab vedotin (Adcetris) Polatuzumab vedotin (Polivy)	Lymphoma
	Anti-CD38 Anti-SLAMF7	Daratumumab (Darzalex) Elotuzumab (Empliciti)	Multiple myeloma
	Anti-RANKL	Denosumab (Xgeva)	Sarcoma

*Immunotherapies are currently emerging in the landscape of treatments for most type of cancers. Among them, a few cancer vaccines are approved, and many more are currently under assessment in clinical trials. List taken from the Cancer Research Institute (CRI) website on July 2019 (www.cancerresearch.org/immunotherapy).*

Among these immunotherapies, vaccines aim at reducing cancer occurrence by preventing cancer-causing infections, in the case of prophylactic vaccines, or at developing strong host immune reactions and subsequent immune memory to efficiently eradicate primary tumor cells and metastasis, in the case of therapeutic vaccines. Therapeutic vaccines hold great promises for long-term remission in patients, in that they can install immunological memory directed against the tumor. Unfortunately, despite extensive research, only four cancer vaccines have made it into the clinic to date: two prophylactic ones, the human papilloma virus vaccine and the hepatitis B virus vaccine for prevention of cervical and liver cancer respectively, and two therapeutic ones, namely the Bacillus Calmette-Guérin (BCG) vaccine, which reduces relapse and metastasis of early stage bladder cancers, and Sipuleucel-T, a cell-based vaccine for advanced prostate cancer (DeMaria and Bilusic, 2019). Therefore, important additional efforts are required to achieve the high expectations of especially therapeutic cancer vaccines at the bedside.

Indeed, the next generation of therapeutic cancer vaccines will necessitate improvements both in terms of vaccine compositions and delivery strategies. In this review, we will discuss how such improvements could be achieved via materials engineering. Furthermore, while vaccination can lead to toxicity concerns due to strong systemic activation of the patient’s immune system, we will present how engineering of targeting materials can enhance the safety profile of vaccines by localizing their effects to specific sites. Therefore, here we aim at providing the reader with design considerations, current challenges and lines of thoughts for the development of potent and safe site-specific targeting therapeutic cancer vaccines. Because very few of these vaccines have been developed to date, this review will take selected examples of targeting cancer immunotherapies – not only vaccines – to highlight possible engineering strategies that can be further applied to vaccination.

## Which Type of Immune Reactions Should Therapeutic Cancer Vaccines Induce?

Engineering an optimal therapeutic cancer vaccine requires a good understanding of the type of immune reactions needed to eradicate tumors. Ideally, the vaccine should elicit potent immune responses that specifically recognize and eliminate all tumor cells present in the body, including those in the primary tumor, in circulation and in metastatic lesions. These immune responses should therefore be cancer cell antigen-specific to limit unwanted systemic side effects and prevent adverse autoimmune reactions. In addition, the vaccine should induce a strong immune memory against the cancer cells, able to efficiently reactivate anti-tumor immunity upon detection of cancer relapse, which is necessary to achieve long-term disease remission. In fact, cancer mortality has been largely imputed to relapses rather than to the primary tumor (Mehlen and Puisieux, 2006). As specificity and memory are hallmarks of the adaptive immune system, therapeutic cancer vaccines aim at activating endogenous cellular or humoral mechanisms of adaptive immunity against tumors.

**FIGURE 1:**
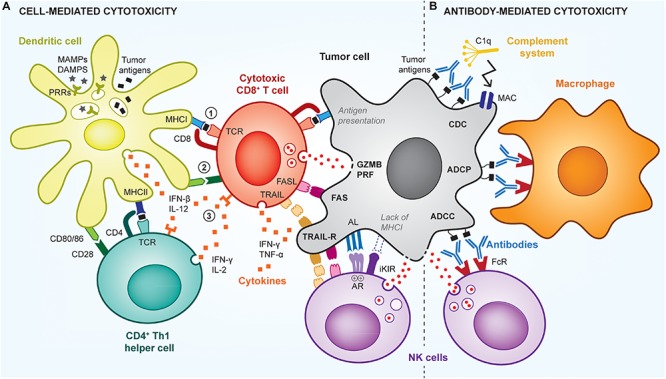
Cell- and antibody-mediated cytotoxic mechanisms of anti-tumor immunity. The immune system uses multiple mechanisms to efficiently kill tumor cells, via cytotoxic CD8^+^ T cells (CTLs), NK cells, or antibody-mediated mechanisms. **(A)** To be activated, T cells need 3 signals from antigen-presenting cells (APCs; e.g., dendritic cell): signal (1) is the presentation of cancer antigens (black squares) via MHC complexes; signal (2) is the signaling induced by co-stimulatory receptors (e.g., CD80/86), which are expressed by the APCs in the presence of adjuvants (e.g., MAMPs/DAMPs); and signal (3) is the stimulation by pro-inflammatory cytokines. Such cytokines are produced by APCs as well as by CD4^+^ Th1 helper cells that further enhance activation of CTLs. Upon activation and recognition of tumor cells, CTLs can induce their death via various pathways, including perforin (PRF)/granzyme B (GZMB), FasL-Fas, TRAIL or inflammatory cytokines. Using similar pathways, NK cells can kill cancer cells that have downregulated their MHCI expression and fail to signal through the inhibitory killer immunoglobulin-like receptor (iKIR), or that overstimulate NK activating receptors (AR). **(B)** Tumors cells can also be targeted by antibodies that induce direct killing via the activation of the complement cascade, through a mechanism called complement-dependent cytotoxicity (CDC), which leads to the formation of membrane attack complexes (MAC) perforating the tumor cell membrane. In addition, antibodies can signal via Fc receptors (FcR) on innate immune cells, to induce antibody-dependent cell phagocytosis (ADCP) of tumor cells by macrophages, or antibody-dependent cell cytotoxicity (ADCC) by NK cells or neutrophils.

The most common strategy exploited for the development of therapeutic cancer vaccines relies on the generation of endogenous cancer-specific cytotoxic CD8^+^ T cells (cytotoxic T lymphocytes, CTLs), due to their unique ability to kill cancer cells upon specific recognition (Farhood et al., 2018). This recognition is mediated by the T cell receptor (TCR) of CTLs that can bind to cancer antigen epitopes mounted on the major histocompatibility complex class I (MHCI) displayed at the cancer cell surface. TCR-signaled CTLs can then induce cancer cell death via multiple pathways, including by degranulation, which releases perforin (PRF)/granzyme B (GZMB), or by upregulation of FasL or TRAIL that signal cancer cell apoptotic pathways (Figure 1A).

To become effective, CTLs need to be educated by antigen-presenting cells (APCs) prior to cancer cell recognition, the most professional APCs being dendritic cells (DCs) and especially CD103^+^ migratory DCs. APCs provide CTLs with three required activation signals, namely (1) the cancer antigen epitope mounted on MHCI, (2) co-stimulatory molecules such as CD80/86 and CD40, and (3) pro-inflammatory cytokines such as IL-12, IFNβ and TNFα (Locy et al., 2018). In addition, activation and survival of CTLs is further supported by activated CD4^+^ T helper (Th) cells via the secretion of additional cytokines, as well as via a process known as “DC licensing,” by which a CD4^+^ Th cell activates a DC through interaction with CD40, and subsequently supports CTLs that come in contact with the same DC (Ridge et al., 1998; Laidlaw et al., 2016). CD4^+^ Th cells are activated by APCs similarly to CD8^+^ T cells, with the exception that the cancer antigen epitope is presented on MHCII instead of MHCI.

Upon activation, CTLs and CD4^+^ Th cells acquire particular phenotypes, which strongly determine the subsequent efficacy of CTL cytotoxic responses (Thorsson et al., 2018). Commonly, phenotypes of CTLs are defined by the cocktail of cytokines they produce, as well as by the cytotoxic pathway they use to induce cell death. Many studies have shown that the production of IFNγ and TNFα by CTLs correlates with better control of tumor burden and enhanced patient survival (Matsushita et al., 2015; Bhat et al., 2017). In parallel, other studies have demonstrated an increased patient survival when CD4^+^ Th cells acquire a Th1 phenotype, characterized by the secretion of IFNγ, TNFα and IL-2. Although more controversial (Chen and Gao, 2019), it has been shown that combination of the Th1 response with a Th17 orientation, characterized by IL-17 production, can be further beneficial (Punt et al., 2015; Thorsson et al., 2018). Noteworthily, not only the type of secreted cytokine matters, but also their variety and amount. Indeed, T cells that secrete multiple cytokines are known to be more efficient than those expressing a single one (Seder et al., 2008). In addition, because each T cell has a unique TCR able to recognize only a single antigenic epitope, immune responses that generate a broad clonality of anti-tumor T cells (i.e., multiple T cells clones) are more robust (Thorsson et al., 2018). All that being said, the ideal type of immune response sought upon vaccination might vary between cancers.

In addition, antibody-mediated cytotoxic mechanisms can also efficiently control tumor growth (Zahavi et al., 2018) and be harnessed by cancer vaccination (Figure 1B). Particularly, antibodies that specifically bind to cancer cells can trigger their elimination by antibody-mediated cellular cytotoxicity (ADCC), antibody-mediated cellular phagocytosis (ADCP) or complement-dependent cytotoxicity (CDC) (Almagro et al., 2018). To date, such mechanisms have been mostly exploited in passive cancer immunotherapies via the infusion of therapeutic antibodies in patients, rather than in the context of humoral-based cancer vaccines, which aim to activate endogenous host anti-cancer antibody responses (Huijbers and Griffioen, 2017). In both cases, antibodies recognize short conformational or linear epitopes exposed on the cancer cell surface (Bayrami et al., 2016; Tarek et al., 2018). Then, innate immune cells, mostly natural killer (NK) cells, macrophages and neutrophils, can detect bound antibodies via their Fc receptors and subsequently induce cell lysis, in case of ADCC, or phagocytosis, in case of ADCP. In contrast, CDC is independent of immune cells and can directly activate the complement pathway to form cytolytic pores in cancer cell membranes, inducing their death. Such antibody-mediated anti-tumor responses are antigen-specific and can provide immune memory if the tumor-specific antibodies are produced endogenously.

Finally, the anti-cancer adaptive immune response sought by cancer vaccination can be further supported by co-activation of other mechanisms of innate immunity. For example, innate lymphoid cells (ILCs), such as NK cells or invariant NK T cells (iNKT), have the ability to control cancer cells in complementary ways to CTLs (Nair and Dhodapkar, 2017; Souza-Fonseca-Guimaraes et al., 2019). For example, NK cells possess cytotoxic capabilities and can lyse cancer cells that downregulate MHCI to avoid T cell recognition or that overstimulate activating receptors on NK cells (e.g., NKG2D, 4-1BB) (Souza-Fonseca-Guimaraes et al., 2019). On the other hand, activation of iNKT cells can result in secretion of Th1 or Th2 cytokines in the microenvironment and increase their CD40L expression. Therefore, iNKT cells can strongly stimulate DC and B cell maturation and indirectly promote T cell responses, thus illustrating a pivotal role in modulating adaptive immune responses (Cerundolo et al., 2009). Nevertheless, because NK or iNKT cells are not antigen-specific and do not establish immune memory, they are often not the primary targets of cancer vaccines.

## What Should Therapeutic Cancer Vaccines Be Composed Of?

The composition of therapeutic cancer vaccines directly relates to their anticipated biological outcomes. Commonly, cancer vaccines are made of antigens that define what is recognized on or within cancer cells, as well as adjuvants that determine the type of immune response that will be induced.

### Cancer Antigens

Including cancer antigens in vaccines is essential to induce targeted cancer cell death as well as to avoid toxic, non-specific immune reactions. Determining the appropriate immunogenic antigen to incorporate in therapeutic cancer vaccines, however, remains extremely challenging. Indeed, the self-origin of cancer cells makes them hard to discriminate from healthy cells, as they carry most of the host proteome, which is naturally immune tolerized to prevent autoimmune reactions. Nevertheless, different types of cancer antigens have been successfully used in cancer vaccines, as detailed below (Vigneron, 2015; Finn, 2017).

First, cancer vaccines often use tumor-associated antigens (TAAs) as targets, which are molecules largely overexpressed in tumor cells (e.g., 10–1000 fold increase in some cases) as compared to healthy cells (Vigneron, 2015). Notably, TAAs can be proteins involved in tumor cell survival and proliferation, such as HER2, EGFR, p53, telomerase, survivin, and Ras, in tumor metabolism, such as folate-related proteins and glucose receptors (e.g., GLUT1), or other proteins such as MUC-1 and mesothelin (Vigneron, 2015). However, the main drawbacks of TAAs reside in the difficulty of inducing strong immunity against them, which must break endogenous immune tolerance mechanisms, while preventing autoimmune reactions against healthy cells.

As an alternative, vaccines can use tumor-specific antigens, which are absent in healthy cells or expressed in limited areas. Particularly, cancer cells can re-express MAGE antigens, NY-ESO-1, 5T4 or the carcinoembryonic antigen (CEA), which are only present during the developmental stage or in very specific tissues (e.g., placenta, testis) in the body (Vigneron, 2015). The restricted expression of such tumor-specific antigens provides the advantage of limiting off-target reactions. Interestingly, it has been shown that some of these antigens are naturally immunogenic and able to raise specific T cell responses in spite of their endogenous origin. Additionally, some types of cancer exhibit specific mutations that can be conserved across patients, such as the BRAF mutation in melanoma, which can constitute good antigenic targets as they are exclusively present in cancerous tissues (Mandalà and Voit, 2013).

Similarly, most tumors contain specific mutated antigens, called neoantigens, due to the higher mutational rate of cancer cells as compared to healthy ones (Schumacher and Schreiber, 2015). These antigens constitute a subclass of tumor-specific antigens, and can be similarly targeted by cancer vaccines. However, the use of neoantigens implies more personalized therapies, as they are different between patients, tumors or even tumor cell subsets. Nevertheless, recent technological advances and ease in genome sequencing allow the fast emergence of such therapies. Neoantigens have been proven to be immunogenic, and their restricted expression in cancer cells highly limits the risk of T cell reactions against healthy self cells. Neoantigens are being widely addressed, with many researchers focusing on understanding how to select, design and deliver the most relevant neoepitopes to incorporate into vaccines (Schumacher et al., 2019).

Furthermore, other strategies use whole cancer cells or cancer cell-derived materials as antigenic components in vaccines, instead of selecting single or combinations of defined antigens (Vermaelen, 2019). Such approaches are particularly interesting as they allow vaccination against multiple cancer antigens, while bypassing the need of identifying them. For example, lethally irradiated cancer cells derived from the primary tumor have been used to induce effective polyantigenic anti-tumor immune responses (Vermaelen, 2019). In addition, tumor cells can be prepared as lysates (González et al., 2014). In such cases, it is expected that the immunogenic responses will be mainly directed against cancer-specific antigens rather than against co-delivered endogenous proteins, since immune tolerance mechanisms would dampen responses to self-antigens. Lastly, cancer antigens have been shown to be present on tumor-derived extracellular vesicles, such as exosomes, as well as on tumor apoptotic debris, which can then also serve as antigenic materials (André et al., 2002). Importantly, targeting multiple antigens provides the advantage of reducing the risk of tumor immune escape, a mechanism by which cancer cells downregulate targeted antigens, mutate them or limit their presentation on MHC to avoid recognition and killing by CTLs.

Finally, cancer vaccines could also be rationally designed to target the tumor *in vivo* and use it as an *in situ* source of cancer antigens, as further discussed in the section “Rationale for Site-Specific Targeting of Therapeutic Cancer Vaccines”. Because these tumor-targeting vaccines can be composed of only adjuvants (i.e., without added antigens), whether it is classified as a therapeutic vaccine or as another type of immunotherapy is arguable.

### Immune Adjuvants

The delivery of antigens alone may induce immune tolerance rather than activation. As a consequence, vaccines need to combine antigens with adjuvants, which are immunostimulatory molecules able to skew immune cells toward the desired type of immune response. Adjuvants can be derived from microbes, so called microbial-associated molecular patterns (MAMPs) or pathogen-associated molecular patterns (PAMPs), from endogenous danger signals released upon cell damage or immunogenic cell death, known as damage-associated molecular patterns (DAMPs), or can simply be cytokines that are naturally secreted to support endogenous immune responses (Tovey and Lallemand, 2010; Tang et al., 2012).

Both MAMPs and DAMPs are able to generate Th1 and CTL immune responses, as mostly intended in cancer vaccines, via the activation of pattern-recognizing receptors (PRRs) on APCs (Tang et al., 2012). Among these PRRs, Toll-Like receptors (TLRs) have been the most studied, with 6 gathering a significant interest in cancer vaccines, namely TLR-2, -3, -4, -7/-8, and -9 (Gay and Gangloff, 2007). These receptors are located in the endosomal compartment of APCs, except for TLR-2 and -4 which are on the cell surface. Consistent with their subcellular location, TLR-3, -7/-8, and -9 primarily recognize nucleic acid ligands from viruses or bacteria, double-stranded RNA, single-stranded RNA and unmethylated CpG oligodinucleotides (ODN), respectively, whereas TLR-2 recognizes bacterial lipoproteins (Lpp) upon dimerization with TLR-1 or -6, and TLR-4 recognizes lipopolysaccharides (LPS) from bacterial outer membranes. Examples of well-known TLR ligands that have been assessed in cancer vaccines are Pam3CSK4 (Zom et al., 2018) and Pam2Cys (Zhou et al., 2019) for TLR-2/1 and -2/6 respectively, poly(I:C) for TLR-3 (Ammi et al., 2015), LPS and monophosphoryl lipid A (MPLA) for TLR-4 (Cluff, 2010), imiquimod and other imidazoquinolines for TLR-7/-8 (Dowling, 2018), and CpG-B for TLR-9 (Shirota et al., 2015). Although these TLR agonists are very potent in activating immune responses, they can be associated with toxicity, which affects their clinical translation. Interestingly, some endogenous extracellular proteins have also been identified as TLR agonists and might be potentially safer considering their endogenous origin. For instance, the extra domain A (EDA) of fibronectin, a matrix protein, can bind to TLR-4 upon proteolytic cleavage and has showed some promises as adjuvant in cancer vaccines in pre-clinical models (Lasarte et al., 2007; Julier et al., 2015).

In addition to TLRs, other PRRs can be targeted by cancer vaccines. For example, the cytosolic DNA sensor cGAS detects aberrant concentrations of DNA in the cytosol and triggers the simulator of interferon genes (STING) pathway (Li et al., 2019). Another example is the cytosolic RNA sensor RIG-I that detects particular viral dsRNA (Tang et al., 2012; Elion and Cook, 2018). Stimulators of these cytosolic nucleic-acid sensor pathways are currently being explored as adjuvants for cancer immunotherapies.

Upon PRR signaling, APCs undergo maturation, which results in increased antigen presentation, expression of co-stimulatory receptors and secretion of cytokines, thus providing the three signals necessary for T cell activation, as previously detailed. Additionally, the nature of the co-stimulatory receptors and cytokine expression by APCs depends on the type of delivered adjuvants. Interestingly, it has been shown that secretion of IFNα and IFNβ by APCs upon maturation can induce direct inhibitory effects on tumor cell proliferation and activate their apoptotic pathways, inducing cancer cell death (Apelbaum et al., 2013).

Since cytokines themselves can strongly support immune responses, they have also been considered as adjuvants in cancer vaccines. Particularly, cytokines can be delivered to promote activation of immune cells, recruit them at specific sites, or induce their proliferation. For instance, IL-2, IL-12, IFNα, and IFNβ have been used to increase survival and activation of T cells, NK cells and APCs. Despite being very effective in boosting anti-tumor immune responses, these cytokines suffer from toxicity-related issues, similarly to TLR agonists, and require further development of appropriate delivery systems to harness their potential in the clinic. On the other hand, chemokines – a subset of cytokines – have been used to attract APCs at the vaccine site, thus enhancing overall antigen presentation and subsequent immune cell activation. While some chemokines induce the recruitment of multiple types of APC (e.g., DCs, macrophages), such as CCL3 and CCL4 (Nguyen-Hoai et al., 2016; Allen et al., 2018), some others recruit specific APC subsets. For example, the delivery of XCL1 specifically attracts the CD103^+^ DCs (Russell et al., 2007; Sánchez-Paulete et al., 2018), known to express the cognate receptor XCR1 and be highly efficient in generating CTLs. Moreover, chemokines can also be used to recruit T cells, rather than APCs. Notably, CXCL10 and CXCL11 have been delivered to increase infiltration of activated T cells in tumors (Groom and Luster, 2011). Lastly, as an alternative to recruitment, *in situ* proliferation of immune cells can be promoted by the delivery of growth factors. Particularly, granulocyte-macrophage colony-stimulating factor (GM-CSF) has been used to expand DC populations in a therapy called GVAX (Simons and Sacks, 2006).

Interestingly, the secretion of multiple cytokines by activated iNKT cells can also be exploited as an adjuvant in cancer immunotherapies, including vaccines (Wolf et al., 2018; Fujii and Shimizu, 2019). Upon activation by CD1d-bound α-Galactosylceramide (α-GalCer; KRN7000) on APCs, iNKT cells secrete large amounts of IFNγ and IL-4 that enhance DC maturation and subsequent antigen-specific T cell responses.

Finally, in addition to exogenous adjuvant delivery, another important strategy in cancer vaccines is to exploit the release of endogenous DAMPs by the tumor itself to self-adjuvant vaccines (Hernandez et al., 2016). Indeed, induction of immunogenic cancer cell death by current therapies, such as chemotherapy, radiotherapy, and/or immunotherapy, can substantially increase the release of DAMPs from dying tumor cells, such as heat-shock proteins (HSPs), adenosine-triphosphate (ATP) or the high mobility group protein B1 (HMGB1) (Tang et al., 2012). Along with endogenous DAMPs, immunogenic cancer cell death co-releases cancer antigens, together promoting antigen spreading, a complex mechanism by which immune reactions are mounted against antigens that were not originally targeted by a therapy (Gulley et al., 2017). As a consequence, any method capable of killing cancer cells in an immunogenic way can potentially boost the effects of cancer vaccines and broaden the anti-cancer immune response to multiple antigens.

## How to Engineer Targeting Materials for Therapeutic Cancer Vaccine Delivery?

Once the cancer vaccine components have been defined, the way they are delivered will significantly impact overall efficacy and safety. As a consequence, the vaccine delivery needs to be rationally designed from the entry into the patient to its terminal effect; this includes the administration route into the patient, the targeting to correct tissues, cell types, subcellular locations and specific receptors, and ultimately the onset of appropriate immune responses. All together, these steps constitute the pharmacokinetic and pharmacodynamic profiles of the vaccine and can be fine-tuned by the use of materials. Here, we will discuss how materials can be engineered to optimally deliver its components and target them into relevant sites.

### Where to Target Therapeutic Cancer Vaccines?

#### Possible Delivery Routes for Therapeutic Cancer Vaccines

From a clinical point of view, cancer vaccines can be conveniently administered to patients via intradermal, subcutaneous, intramuscular, intravenous or intratumoral routes, if the tumor is easily accessible at the body surface, as in skin cancers. However, intratumoral administration can become challenging depending on the tumor size, location, the number of tumors to inject, as well as on the intrinsic heterogeneity of tumor structures that can lead to non-homogenous drug distribution (Marabelle et al., 2018). Similarly, intralymphatic, intranodal and intrasplenic delivery routes are relatively complex, although they may be relevant from a biological point of view, as discussed below. Other routes, such as topical, oral or intranasal, are often less utilized yet might be appropriate in specific types of cancer.

The choice of the vaccine delivery route should be based on the anticipated biological mechanisms of action, since its efficacy depends on its bioavailability at the targeted sites. In the case of therapeutic cancer vaccines, tumor tissues and lymphoid organs are generally considered as the most interesting sites to target, and many direct or indirect strategies have been explored to deliver drugs at these locations.

#### Rationale for Site-Specific Targeting of Therapeutic Cancer Vaccines

**FIGURE 2:**
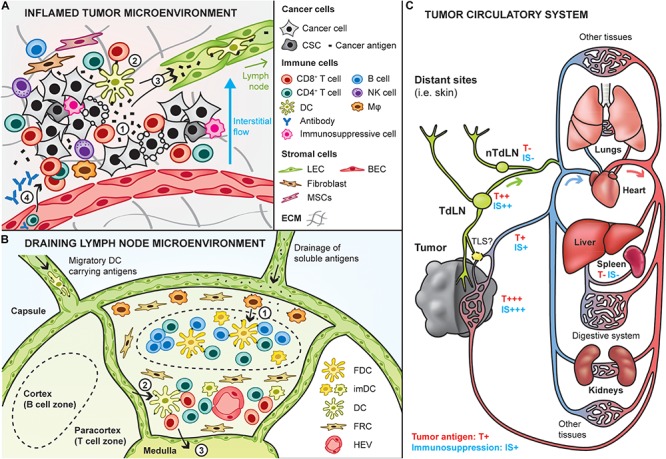
Site-specific targeting of therapeutic cancer vaccines. Cancer vaccines can be designed to specifically target sites that are potent for inducing anti-tumor immunity, including the tumor and lymphoid tissues. **(A)** Interactions between the tumor and immune infiltrates in the inflamed tumor microenvironment. Upon cancer cell death (e.g., T cell-mediated), cancer antigens are released in the local environment (1) and can be taken up by dendritic cells (DCs) to induce *in situ* activation of cancer-specific T cells (2). In addition, cancer antigens are either passively drained by lymphatic vessels or actively transported by immune cells trafficking to the draining lymph node (3), where potent immune responses can be induced. Then, T cells activated in the lymph nodes can home into the tumor to kill tumor cells (4). In addition, humoral immune responses may be triggered in the lymph node and can lead to the production of cancer-targeting antibodies that enter the tumor bed to induce antibody-mediated cytotoxic mechanisms (4). It is important to note here that the depicted tumor-immune interactions do not take place in tumors that are known as immune deserts (i.e., non-inflamed). CSC: cancer stem cells, Mφ: macrophage, LEC/BEC: lymphatic/blood endothelial cell, MSC: mesenchymal stem cells, ECM extracellular matrix. **(B)** Lymph nodes are relevant to target by therapeutic cancer vaccines as they are naturally optimized to induce strong immune responses, due to their high content in immature DCs, and naive T and B cells. Naive lymphocytes enter the lymph node via the high endothelial venules (HEV). Antigens and adjuvants can enter lymph nodes in soluble form and be further transported by subcapsular macrophages to follicular DCs to induce B cell responses (1). In addition, migratory DCs loaded with cancer antigens can go into the paracortical zone (2), where they activate antigen-specific CD4^+^ and CD8^+^ T cells. Activated T cells exit the lymph node via the medulla (3). FDC: follicular dendritic cell, imDC: immature dendritic cell, FRC: fibroblastic reticular cells. **(C)** Overview of the tumor circulatory system and tumor antigen biodistribution. Tumors are connected to the blood circulatory system by blood vessels (arterial system in red, venous system in blue) and lymphatic routes (in green). The tumor is a relevant site to target by cancer vaccines as it has the highest concentration of tumor antigens (high exposure: T+++), although its high immunosuppression (IS+++) might impair vaccine efficacy. Alternative sites to target can be the tumor-draining lymph node (TdLN) also relatively highly exposed to tumor antigens. As they are less immunosuppressed, targeting non-tumor-draining lymph nodes (nTdLN) and the spleen might lead to better immune activation upon vaccination, yet their poor exposure to tumor antigens might require the use of exogenous cancer antigens.

Tumors are the primary location where therapeutic efficacy is sought, when they cannot be fully removed by surgery. Accordingly, targeting cancer vaccines into tumors is an appealing strategy to induce direct *in situ* cytotoxic effects and promote potent antigen-specific adaptive immune responses (Marabelle et al., 2014). Of particular importance, the tumor is the main source of cancer antigens, and thus can be used in place of or in combination with antigens from the vaccine (Figure 2A). Because the tumor gathers high concentrations of all cancer antigens at the same location, it theoretically constitutes an ideal target to promote broad polyantigenic immune responses. Interestingly, targeting the vaccine into tumors may also allow induction of immune reactions against antigens expressed only by small subpopulations of cancer cells, such as cancer stem cells, which are particularly important to eradicate (Saygin et al., 2019). Another advantage of targeting tumors *in vivo* is provided by the local release of antigens and DAMPs upon intratumoral cytotoxicity, which can enhance antigen spreading and thus the vaccine’s effects, as discussed in the section “Immune Adjuvants” (Hernandez et al., 2016; Gulley et al., 2017). Finally, some inflamed tumors are the battlefield of pre-existing anti-tumor immune reactions, which can be further supported or re-activated *in situ* by tumor-targeting vaccines.

On the other hand, tumor-targeting strategies also present some important limitations for vaccination. First, although intratumoral activation of immune cells has been demonstrated, tumors are not physiologically optimized to mount strong immune responses, as opposed to lymphoid tissues (Thompson et al., 2010). Secondly, the high heterogeneity of tumor compositions may strongly affect the efficacy of the vaccines, possibly requiring tailoring per tumor characteristics (Binnewies et al., 2018). In particular, vaccines targeted to tumors that are known as immune deserts (i.e., lack of immune infiltrates) are likely to be poorly effective as compared to targeting into inflamed tumors. Thirdly, the tumor microenvironment is known to be strongly immunosuppressive, which would undeniably prevent potent anti-tumor immune reactions to be mounted upon vaccination.

To overcome these limitations, other sites might be interesting to target by cancer vaccines, notably lymphoid organs, including lymph nodes and the spleen, which are physiologically optimized to build potent immune responses and might be exposed to tumor antigens (Thomas et al., 2014; Rotman et al., 2019; Figure 2B). Indeed, tumor interstitial fluid and debris are drained from the tumor to the lymph nodes through lymphatic vessels, and then to the blood systemic circulation. In addition to lymphatic routes, some debris can directly enter the blood circulation via tumor venous drainage. Eventually, they are filtered by the spleen, liver and kidneys (Figure 2C). Recently, it has been highlighted that tertiary lymphoid structures (TLS) can form in proximity of tumors, which might constitute another relevant site to target, although our current knowledge on these structures remain limited to date (Sautès-Fridman et al., 2019).

Targeting the tumor-draining lymph nodes (TdLNs) might therefore constitute a good alternative to direct tumor-targeting, considering their high exposure to cancer antigens, optimal content and organization of immune cell populations and conserved structures, which would permit the development of more generic (i.e., less tumor-specific) cancer vaccines (Jeanbart et al., 2014). Nevertheless, TdLNs can also be affected by tumor-derived immunosuppression, as they drain immunosuppressive factors from the tumor. In addition, tumor-draining lymph nodes are sometimes surgically removed for diagnosis purposes, to establish the metastatic and aggressiveness profile of cancers.

Should this be the case, targeting cancer vaccines to the spleen or to non-tumor draining lymph nodes (nTdLNs) remain other relevant options (Jeanbart et al., 2014). Since those are located downstream in the circulatory system, they are less exposed to the tumor immunosuppression, although also less supplied with tumor antigens. As a consequence, adding exogenous tumor antigens in vaccines targeting these sites might be necessary to achieve proper efficacy.

Practically, tumors can be directly targeted via intratumoral delivery or indirectly by the use of tumor-targeting technologies. On the other hand, lymph nodes can be indirectly targeted by delivering the vaccine in the tissues they drain, via topical, intradermal, subcutaneous or intramuscular routes, for example, which might be more convenient than via intranodal or intralymphatic injections. Lastly, the spleen can be efficiently targeted via intravenous perfusion, or less commonly via an intrasplenic route.

### Passive Targeting Using Material Engineering

Establishing the vaccine delivery route and strategy will inform on the intrinsic properties required for the vaccine to be efficient, notably providing criteria on the components’ half-life, stability, solubility, toxicity or biodistribution. Modulation of these parameters has been widely achieved by the use of materials. In addition, some materials can act themselves as immunostimulants (Sun et al., 2017), as targeting tools (Weissleder and Pittet, 2008), or have direct cytotoxic effects on cancer cells (Zou et al., 2016), further enhancing the vaccine outcomes.

#### Choice of Material Physicochemical Properties

When developing a new material for cancer vaccines, or using an already existing one, the choice of material primarily depends on its physicochemical properties, such as its size, shape, charge, solubility and elasticity. These parameters will affect the vaccine by modifying its biodistribution, cell internalization and activation capabilities, as well as its half-life and release kinetics. Thus, the material needs to be chosen according to both the delivery route into the patient and its ability to target and get metabolized by the correct cell types.

##### Size

First, the size of a material, or particle, can range from a few nanometers up to several microns and will influence its drainage, biodistribution, which cells internalize it as well as its retention time. It has been demonstrated that intradermal injection of nanoparticles ranging from 20 to 200 nm can enter the lymphatic system and drain to the lymph node, with a preference for particles ranging around 40 nm, whereas larger ones will be retained at the injection site and internalized by APCs before being transported to the lymph node via cellular trafficking (Swartz, 2001; Reddy et al., 2006; Irvine et al., 2013). On the other hand, if the formulation is injected intravenously, carriers smaller than 5 nm will not only be filtered by the kidney in less than 5 min but will also escape the vessels to diffuse in the neighboring tissues, whereas larger particles have a longer half-life in the blood (Choi et al., 2011; Hoshyar et al., 2016). Interestingly, compared to the tight junction of the endothelium of blood vessels in healthy tissues (5–10 nm), fast growing cancer vessels have looser junctions with pores ranging from 200 to 1200 nm, allowing particle extravasation within that range (Chauhan et al., 2012). Upon extravasation, particles can additionally be retained in the tumor for extended time, as a result of impaired tissue drainage. This is a process known as the enhanced permeability and retention (EPR) effect that has been widely exploited in cancer animal models for the development of tumor targeting nanosystems, but that remains controversial for use in humans (Danhier, 2016; Golombek et al., 2018). Unfortunately, although larger particles have several advantages when injected directly in the bloodstream, their penetration efficiency in the tumor is reduced compared to smaller particles (Hauert and Bhatia, 2014).

At a cellular level, the size of the carrier influences endocytosis by specific cell types. Indeed, it has been demonstrated that particles of 20–600 nm are preferentially taken up by DCs, whereas particles from 0.5 to 5 μm are rather taken up by macrophages (Xiang et al., 2006; Kanchan and Panda, 2007). Finally, the material fate upon intracellular trafficking will directly affect the efficacy of the vaccine itself since the payload has to reach the correct subcellular compartment to activate the cognate receptors or signaling cascade. Briefly, particles ranging from 250 nm up to 3 μm are preferentially taken up by phagocytosis whereas smaller particles enter the cell through pino- or macro-pinocytosis (Rivolta et al., 2012). Several uptake studies on cancer cells have demonstrated that the highest uptake was observed for particles around 50 nm (Chithrani et al., 2006).

##### Shape

Second, the shape of the material also affects its systemic biodistribution, circulation in blood, cellular uptake and interactions. For example, it has been shown that for some materials, such as gold nanoparticles, rod-shape structures tend to accumulate more in the spleen and less in the liver than their spherical counterparts (Arnida et al., 2011; Black et al., 2014); although not a general rule, this exemplifies how material shape can influence biodistribution. In addition, non-spherical particles in the bloodstream tend to marginate more and escape the blood flow (Toy et al., 2014). Microscopically, it has been demonstrated that the rate of cellular internalization of non-spherical particles depends on their angular orientation relative to the cell membrane (Sharma et al., 2010; Behzadi et al., 2017). Furthermore, spherical particles are favorably internalized by monocytes/macrophages compared to particles with a high aspect ratio, which will marginate and target the endothelial cell and evade macrophage uptake (Peiris et al., 2012). Finally, an interesting study has highlighted that T cell activation is enhanced when using ellipsoidal synthetic APCs rather than spherical ones, due to an increased contact interactions with the immune cell membrane (Meyer et al., 2015).

##### Elasticity

Thirdly, it is hypothesized that the elasticity of particles influences cellular uptake and tumor accumulation properties. Generally, quantum dots, gold or magnetic particles are considered hard particles, whereas hydrogels, liposomes or polymersomes are described as soft particles. Overall, harder particles are better internalized than soft materials (Beningo and Wang, 2002; Anselmo et al., 2015). Anselmo et al. (2015) also showed that soft particles circulate at a higher concentration in the blood at early times after intravenous delivery and were slower and less endocytosed compared to hard particles. In addition, it was demonstrated that soft nanolipogels accumulated more in tumors compared to hard ones (Guo et al., 2018).

##### Charge, hydrophobicity and other chemical properties

Lastly, compared to the parameters discussed above, the chemical properties are based on intrinsic characteristics of the material, such as its charge, hydrophobicity and functional groups. Indeed, the material charge – cationic, anionic or neutral – influences cell internalization, immune activation and blood half-life. Since cell membranes are negatively charged, they will take up positively charged molecules much faster due to electrostatic interactions compared to other particles (Foged et al., 2005). However, the uptake of positively charged particles can disrupt cell membranes, leading to increased cell toxicity (Fröhlich, 2012). Furthermore, several studies have demonstrated that changing the charge of a material from negative to positive can induce a higher immune response (Wen et al., 2016). Finally it has been demonstrated that neutral particles have a slower internalization rate than charged ones (Owens and Peppas, 2006).

With regard to hydrophobic materials, a shorter half-life in the bloodstream is observed compared to their hydrophilic counterparts due to the reticulo-endothelial system recognizing them as foreign and removing them in the liver or the spleen (Owens and Peppas, 2006). In addition, a positive correlation has been demonstrated between hydrophobicity and immune activation (Moyano et al., 2012). However, hydrophobic materials can be “masked” to prevent removal and reduce intrinsic immune activation by coating them with a hydrophilic material such as polyethylene glycol (PEG), for example. Such a strategy is useful to improve the delivery of vaccines with hydrophobic compounds in the particle core (Maiti et al., 2019). Nevertheless, surface modification of particles additionally modulates their half-life and distribution profile in the body.

The interplay of all these parameters and how they affect treatment outcomes shows the importance of thoroughly characterizing new materials and their intrinsic properties *in vitro* and *in vivo*. Beyond that, core material properties can be further tuned by modifying the material with particular molecules, ligands or polymers, to fulfill specific criteria and needs.

#### Selected Examples of Different Types of Material

**FIGURE 3:**
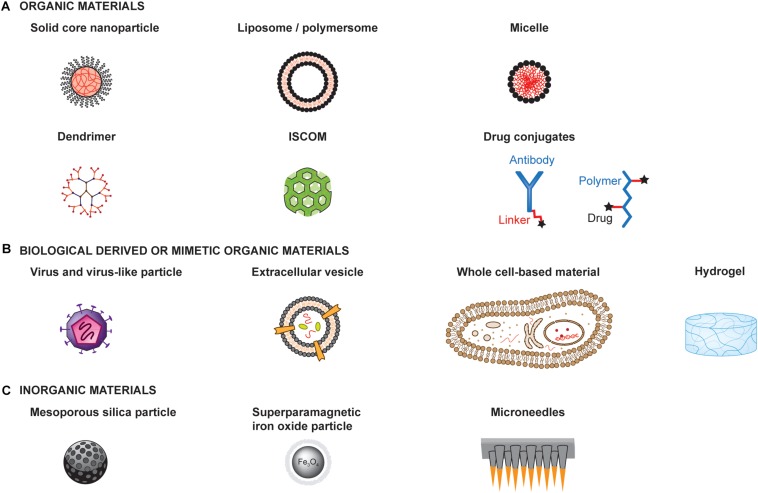
Examples of materials for the development of therapeutic cancer vaccines. Materials can be engineered to enhance the therapeutic efficacy and safety profiles of cancer vaccines. Materials with very different structures and physicochemical properties can be used as a basis for engineering the delivery of adjuvants and antigens to optimize immune activation. Such materials can be organic materials **(A)**, including those derived from or mimicking biological materials **(B)**, or inorganic materials **(C)** ISCOM: Immunostimulatory complex.

The extensive research on material engineering for drug delivery has provided a tremendous amount of available materials and technologies that could be used for the development of cancer vaccines. Here, we present a few examples of different types of materials to illustrate possible designs and structures (Figure 3).

Materials can be organic or inorganic, each having specific properties, advantages and limitations that should guide the choice of a material. Furthermore, organic material can either be synthetic, such as poly(D,L-lactide-co-glycolide) (PLGA), poly(γ-carboxyglutamic acid) (γ-PGA), and PEG, or natural, such as dextran, alginate, lipids and chitosan (Figure 3A). Similarly, they can be synthetically produced or derived from biological origin (Figure 3B). They can form a broad range of structures, including solid core particles, vesicles, micelles, emulsions, dendrimers or hydrogels. On the other hand, inorganic particles have the advantage of having rigid structure, controllable synthesis, with a size range of 2 to 150 nm, as well as low toxicity, although most are not biodegradable. Examples of inorganic particles include silica-based and magnetic particles (Figure 3C).

##### Solid core nanoparticles (NPs)

Nanoparticles are spherical particles with solid cores in the nanoscale size and have been extensively used for drug delivery over the past decades (de Titta et al., 2013; Tran et al., 2017; Ankita et al., 2019). Apart from their spherical shape, most of their parameters and characteristics can be tuned, such as their charges, hydrophobicity or surface properties (i.e., by conjugation of specific moieties), to give a few examples (van der Vlies et al., 2010).

##### Liposomes and polymersomes

Liposomes and polymersomes are 50–500 nm often spherical bilayered vesicles composed of phospholipids or block copolymers, respectively. They can incorporate hydrophobic or viral envelope glycoproteins on their bilayer as well as encapsulate hydrophilic molecule in their core (Senapati et al., 2018). Similarly, to NPs, these vesicles are highly versatile since it is possible to modify most of their parameters, by modifying their surface charge (Mo et al., 2012) or conjugating targeting ligands (Noble et al., 2014), for instance. Interestingly, they can be designed to release their payload in specific subcellular compartments (Jiang et al., 2012).

##### Micelles

Micelles are self-assembled spherical materials composed of amphiphilic block copolymers with a hydrophobic core and a hydrophilic corona (Hanafy et al., 2018). These colloids will spontaneously form at a specific concentration, called the critical micelle concentration (CMC), and temperature. Hydrophobic molecules can be encapsulated into micelles through physical, chemical or electrostatic interactions (Park et al., 2008).

##### Dendrimers

Dendrimers are spherical macromolecules composed of many branches originating from a central point forming a star-like structure. The advantages of these particles are their highly tunable properties since their molecular weight, size, flexibility, branching density, and solubility can be modulated (Tran et al., 2017; Sherje et al., 2018). Interestingly, it is possible to both conjugate dendrimers with several different drugs using different chemistry and “encapsulate” poorly water-soluble molecules into them (Wang et al., 2011). Furthermore, if the polymer used in the dendrimer is positively charged, DNA or RNA can be complexed to it for delivery into cells (Shan et al., 2012). The main drawback of this material is its potential toxicity, and bio-incompatibility, depending on its surface physico-chemical properties (Palmerston Mendes et al., 2017).

##### Immunostimulating complex (ISCOM)

Immunostimulating complexes are cage like particles of 40 nm composed of phospholipids, cholesterol, saponin adjuvant Quil A and protein antigens (Homhuan et al., 2004). Usually the antigen is not directly conjugated to the particle but rather interacts by hydrophobicity (Peek et al., 2008). In addition, they naturally induce an immune response, thus acting as immunostimulant materials.

##### Hydrogels

Hydrogels are a three-dimensional network of hydrophilic polymers cross-linked together. They have the capacity to retain large quantities of fluids and can be chemically modified to insert enzymatic, hydrolytic or stimuli-responsive components to ensure their biodegradability (Peppas et al., 2000). The main advantage of hydrogels is their high water-content similar to biological tissues, thus reducing surface tension induced by the material. In addition, the drug loading and release rate can be tightly controlled by modifying the quantity of gel cross-linking (Lin and Metters, 2006). An interesting feature of hydrogels is the possibility to induce their gelation *in situ* with a specific stimuli such as pH, temperature or light (Van Tomme et al., 2008).

##### Drug-conjugates

These materials simply consist of a drug conjugated to a polymer, or a protein via a linker, which can be cleavable or not. This delivery system has the advantage of reducing treatment toxicity and adverse side effects, solubilizing the drug as well as an easy synthesis (Dan et al., 2018). Cleavable linkers are either acid-sensitive, glutathione-sensitive, lysosomal protease-sensitive or β-glucuronide-sensitive, whereas non-cleavable linkers usually have thioether bonds, which do not have the risk of releasing the drug at the wrong time (Dan et al., 2018).

##### Viruses and virus-like particles

Viruses and virus-like particles both have the advantage of naturally inducing a strong immune response due to their envelope (Zhang et al., 2000). In addition, they can be used as a delivery system for genes, antigens or drugs into tumor cells (Chulpanova et al., 2018). The choice of virus for a treatment will depend on the virus tropism, size and longevity of the desired gene that has to be delivered, as well as on its safety profile. The most common viruses currently tested in clinical trials as oncolytic viruses for cancer therapies are adenoviruses, herpes viruses, measles viruses, retroviruses, vaccinia viruses, and vesicular stomatitis viruses. Despite their relative success with inducing tumor regression, a major drawback of viruses is that they are strongly neutralized by host antibody responses upon re-injection. In addition, the immune response can be diverted from tumor antigens to viral antigens (Cawood et al., 2012). Another option is to use only highly immunogenic virus-like particles (VLP) to induce a strong immune response against tumor antigens without having the issue of self-replication and safety concerns caused by viruses (Cubas et al., 2011; Li et al., 2013; Palladini et al., 2018; Thong et al., 2019). These particles can usually range between 20 and 800 nm (Pushko et al., 2013).

##### Extracellular vesicles (EVs)

Extracellular vesicles are biological materials naturally secreted by cells and delimited by a lipid bilayer, commonly found with a size of 20–500 nm, although some can reach several microns (van Niel et al., 2018). They can be derived from the cell plasma membrane in case of microvesicles or from endosomal origin in case of exosomes. As important mediators of intercellular communication, extracellular vesicles can carry proteins, nucleic acid, metabolites and lipids from one cell to another. As such, they have raised interest for possible use as drug delivery systems (Vader et al., 2016). Interestingly, it has been shown that the composition of extracellular vesicles can be modified by engineering either the producing cells or the vesicles after isolation. Although EVs are considered poorly immunogenic carriers (Saleh et al., 2019), they play a role in mediating immunostimulating or immunosuppressive responses (Robbins and Morelli, 2014).

##### Whole cell-based materials

Mammalian cells are living materials also delimited by a lipid bilayer, with a typical size of 10–50 μm of various shapes, that can be used as carriers to deliver drugs or as therapeutic agents *per se* when administered into patients (Cheng et al., 2019; Gong et al., 2019). To prevent cell rejection upon delivery, cells for clinical use are often derived from autologous sources, processed *ex vivo* and re-administered into the patient. One key advantage of using living cells as delivery materials is their ability to actively migrate to specific sites and to dynamically interact with endogenous cells and tissues (Leibacher and Henschler, 2016). Nevertheless, controlling the fate of living materials upon delivery can be challenging due to their high complexity.

##### Silica-based nanoparticles

Silica-based nanoparticles (SiNPs), especially porous SiNPs such as mesoporous silica nanoparticles (MSN), are used for drug delivery due to their high loading capacity and the possibility to control the release and encapsulation of different molecular weight drugs (Lai et al., 2003). In addition, MSN can be functionalized with targeting ligands, antibodies, peptides and even magnetic particles (Mamaeva et al., 2009).

##### Superparamagnetic iron oxide nanoparticles

Superparamagnetic iron oxide nanoparticles (SPIONs) are receiving increasing attention due to their broad applications in chemotherapy, hypothermia, magnetic resonance imaging (MRI), cell and tissue targeting, to mention a few (Quinto et al., 2015; Senapati et al., 2018). They are composed of an inner magnetic core and a hydrophilic coating polymer, such as PEG, polysaccharide and poly(vinyl alcohol), which can be used to deliver drugs or conjugate targeting ligand (Laurent et al., 2014). Due to their magnetic properties, studies showed the possibility to use an external magnetic field to localize them in the correct tissue and/or heat them to kill cancer cells.

##### Microneedles

Microneedles are sharp protrusions measuring from 100 μm to less than 1 mm, and are used as topical materials for local drug delivery. They are minimally painful for the patient and can be self-administered. The needle tips can be coated with protein, viruses, drugs or immunotherapy and will release the payload in a controlled slow manner (Ingrole and Gill, 2019). In the context of melanoma, for instance, transdermal delivery of immunotherapies with microneedles has demonstrated promising efficacy (Ye et al., 2017).

### Engineering Tumor-Targeting Materials

In addition to their intrinsic physicochemical properties, materials can be further engineered to preferentially or specifically target tumors. Until recently, tumor-targeting materials have been primarily developed to deliver immunotherapeutic or chemotherapeutic drugs, or for diagnostic purposes, rather than for cancer vaccination. Therefore, we here focus on the different targeting technologies used by cancer immunotherapies in a broader scope, considering that they could inspire the design of future cancer vaccines. Particularly, we detail how tumors can be targeted at different levels, including macroscopic targeting of the tumor environment and microscopic targeting of cancer cells, tumor-associated stromal cells and the tumor extracellular matrix (ECM).

#### Targeting the Tumor Biochemical Environment

Due to unusual metabolism, the tumor environment has unique biochemical properties that differ from those of healthy tissues, and that can be used to activate or release drugs in a stimuli-responsive fashion, for example based on pH, oxygenation, protease contents, and chemokine secretion. Using stimuli-responsive materials, it may be possible to improve drug safety by limiting activity in off-target sites.

##### pH-responsive materials

Due to a high metabolism, the tumor environment is at a pH of 6.5 compared to the physiological one at 7.4 (Tian and Bae, 2012). This decrease in pH is caused by an increase in lactate and hydrogen ions produced to permit the substantial and rapid tumor growth. This pH difference has been exploited to develop materials capable of shrinking, aggregating or even enhancing cellular uptake upon tumor microenvironment entry (Wu et al., 2018). For example, particles coated with a zwitterionic monolayer change charge on their surface from negative to positive upon entering the tumor thus enhancing cell uptake and aggregation (Mizuhara et al., 2015). Another option to induce aggregation or release would be to have acid-labile amide bond breakage (Wu et al., 2018). Such strategies have been used to deliver chemotherapy (Yang et al., 2017), thermal therapy (Liu et al., 2017) or for tumor imaging (Hoffmann et al., 2012).

##### Hypoxia-responsive materials

A well-known characteristic of tumors is their low content of oxygen (Shannon et al., 2003; Bennewith and Dedhar, 2011). Based on this property, engineers have developed materials that incorporate bioreductive linkers, such as nitroimidazole analogs, thiol groups, and azobenzene moieties to deliver drugs upon entry into tumors (Guise et al., 2014; Ahmad et al., 2016; Kulkarni et al., 2016). For example, hypoxia-responsive nanoparticles have been developed to release doxorubicin in squamous carcinomas (Thambi et al., 2014). Similarly, prodrugs have been engineered to be activated in low-oxygen environments (Hunter et al., 2016). However, as hypoxia increases with tumor growth, hypoxia-sensitive drugs may have limited efficacy for early stage tumor targeting.

##### Protease-responsive materials

Many tumors exhibit abnormal enzymatic activity (Anderson and Cui, 2017; Yao et al., 2018), including the overexpression of matrix-metalloproteinases (MMPs) (Gialeli et al., 2010), caspases (Nejadnik et al., 2015), urokinase-type plasminogen activators (uPA) and cathepsins (Joyce et al., 2004). Therefore, including protease substrate sequences in materials and prodrugs has been exploited to specifically release drugs into tumors and limit their side-effects. Furthermore, some materials can change size and shape upon protease exposure, for example forming nanostructures (Hu et al., 2014; Anderson and Cui, 2017). As an example, Tanaka et al. designed a gelator precursor that self-assembles into nanofibers upon exposure to MMP-7 in tumor cells, inducing their death (Tanaka et al., 2015). Another study used caspase-sensitive gold NPs (AuNPs) as an apoptosis-inducing imaging probe (Sun et al., 2010).

##### Chemotaxis-based cellular tumor targeting

Tumor inflammation induces the secretion of chemokines, such as CXCL12, which are able to recruit specific cell types. Based on this mechanism, active tumor targeting can be achieved by the delivery of cells capable of sensing these chemokine gradients and actively migrating into tumor-inflamed regions (Cheng et al., 2019), such as myeloid cells, T cells, neural stem cells and mesenchymal stem cells (MSCs) for example (Leibacher and Henschler, 2016; Combes et al., 2018). Furthermore, these cells can be engineered to deliver anti-cancer drugs; for example, MSCs have been genetically modified to overexpress IFNβ or to carry paclitaxel into tumors (Ling et al., 2010; Sadhukha et al., 2014).

#### Targeting Tumor Cells

**FIGURE 4:**
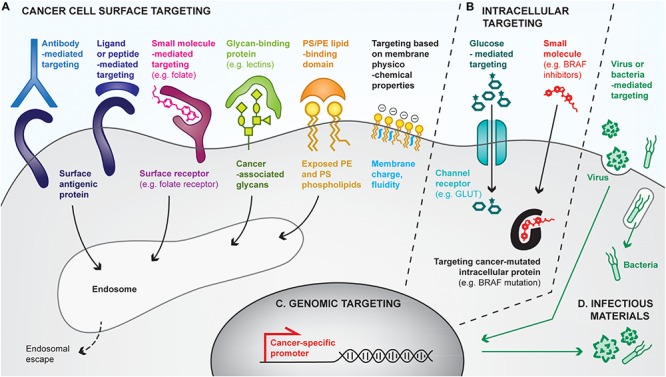
Overview of strategies for cancer cell targeting. Biomolecular engineering can be used to preferentially target the cancer cell at multiple levels, using differences between cancer cells and healthy ones to discriminate between them. **(A)** Cancer cells can be targeted with cell surface-binding moieties, based on specific affinities with cell-surface antigens, receptors, glycans, lipids or based on physicochemical properties (e.g., membrane charges). Most cell surface targeting strategies will lead to endocytosis of the targeting moiety. **(B)** The cancer cell cytoplasm can be directly targeted by using channel receptors that transport small molecules, or by using small molecules capable of crossing cell membranes. **(C)** Cancer cells reactivate specific promoters that are silenced in healthy cells, allowing cancer cell targeting by the delivery of genes placed under cancer specific promoters. **(D)** Finally, some pathogens (e.g., oncolytic viruses, bacteria) favorably infect and replicate in cancer cells, often leading to their death, thus providing additional means for preferential cancer cell targeting.

As cancer cells are the ones to eradicate, they constitute the ultimate target of cancer immunotherapies, including vaccines. Currently, many clinical treatments use targeted therapies to directly kill tumor cells. Coupling such targeting strategies with immune adjuvants would be valuable to turn them into therapeutic cancer vaccines, thus colocalizing tumor cell antigens and immunostimulatory molecules. Cancer cells can be targeted at multiple levels, including cell surface, intracellular or genomic levels, or by other approaches that use infectious materials (Figure 4).

##### Targeting the cancer cell surface (Figure 4A)

One of the most common approaches to target cancer cells relies on affinity-based interactions of surface tumor-associated antigens with antibodies or antibody derivatives (e.g., Fab, scFv). As a clinical example, HER2-positive tumor cells can be targeted by intravenous or subcutaneous injection of anti-HER2 antibodies, which accumulate at the cancer cell surface due to their high specific affinity for the receptor, in both the primary tumor and metastases. Such antibodies can display intrinsic activities to affect tumor cell growth, notably by blocking the surface protein functions and by triggering antibody-mediated cytotoxicity. They can additionally be modified with anti-cancer drugs or adjuvants for the development of cancer vaccines (Hong et al., 2011). For example, Sharma et al. (2008) have chemically conjugated anti-HER2 to CpG, which has led to tumor eradication and induction of protective memory when combined with anti-GITR immunotherapy. In addition, tumor-targeting antibodies have been conjugated to material surfaces, such as nanoparticles (Kubota et al., 2018) or liposomes (Espelin et al., 2016), to confer them the ability to target tumors. Interestingly, biological cell-based materials can be similarly engineered; for instance, in the context of chimeric antigen receptor (CAR)-T cell therapies, patient-derived T lymphocytes are transduced with a modified TCR that comprises a scFv fragment recognizing a specific cancer antigen (Jackson et al., 2016). Although not a cancer vaccine, CAR-T cell therapies strongly mimic their purpose, by both delivering tumor-specific cytotoxic T cells and having the potential to establish anti-tumor memory (McLellan and Ali Hosseini Rad, 2019).

In addition to antibodies, cell surface receptors can be targeted using receptor ligands, peptides, or small molecules. Indeed, ligand/receptor interactions have been exploited to target the epidermal growth factor receptor (EGFR) on urinary bladder cancer, by delivering an EGF-diphteria toxin fusion protein, for example (Yang et al., 2013). Alternatively, cancer-targeting peptides can be found by phage display screening (Zhang et al., 2001), and have the advantages of being smaller and potentially easier to synthesize than antibodies. Lastly, small molecules have been used to functionalize materials to target cancer cell receptors. Particularly, materials conjugated to folate can successfully bind to cancer cell folate receptors with very high affinity (Xia and Low, 2010; Tao et al., 2015).

Not only proteins can be targeted at the cancer cell surface, but also glycans or lipids. Particularly, cancer cells express specific glycans or overexpress others as compared to healthy cells (Dube and Bertozzi, 2005), which can be targeted using glycan-binding proteins, notably lectins. For example, the conjugation of the specific rBC2LC-N lectin to bacterial exotoxin has shown successful targeting and therapeutic effects in pancreatic cancer (Shimomura et al., 2018). Interestingly, lectins themselves can induce autophagy or apoptosis of cancer cells (Yau et al., 2015). Similarly, cancer cells lack the ability to maintain the natural lipid asymmetry in cell membranes, thus exposing phosphatidylserine (PS) and phosphatydilethanolamine (PE) on the outer leaflet of their membranes, has encouraged the development PS/PE-targeting drug delivery systems (De et al., 2018).

Finally, differences have been found in the physicochemical properties of cancer cell membranes, which are more negative and more fluid, as compared to healthy cells (Bernardes and Fialho, 2018). Approaches targeting such differences have been attempted (Chen et al., 2016a), but are likely to be less efficient than those relying on specific interactions for drug delivery purposes.

##### Targeting the cancer cell cytosol (Figure 4B)

Some strategies have been developed to target the cancer cell cytoplasm, for example by using molecular transport via specific channel receptors. Particularly, many cancer cells overexpress the GLUT1 glucose channel receptor to increase their glucose metabolism. Conjugation of glucose to small molecules enables their transport through GLUT1 into the cytoplasm, as exemplified by Glucosfamid or glucose-conjugated paclitaxel (Liu et al., 2007; Calvaresi and Hergenrother, 2013). Interestingly, conjugation of glucose to larger moieties, such as nanoparticles, has allowed an increase of their uptake by cancer cells, yet via clathrin-dependent endocytosis (Dreifuss et al., 2018).

Another approach that can be considered as cytoplasmic targeting is the delivery of small molecules that passively diffuse through cell membranes, but that mostly display activity in cancer cells upon binding to their cytoplasmic target. For example, BRAF inhibitors selectively target the BRAF^V600E^ mutated protein present in melanoma cells but not in healthy ones (Sharma et al., 2012; Karoulia et al., 2017). Similarly, prodrugs can be engineered to be activated in the cytosol of cancer cells specifically (Zhang et al., 2017b).

##### Targeting the cancer cell genome (Figure 4C)

Cancer cells additionally upregulate some specific promoters that can be targeted for gene delivery. For example, the telomerase hTERT promoter has been shown to be re-activated in 90% of human cancers while being silenced in healthy cells (Jafri et al., 2016). Delivering genes under the control of such promoters allows restriction of their expression to cancer cells (Zarogoulidis et al., 2013). Such approaches have mostly been used to deliver cytotoxic genes, such as suicide genes inducing cancer cell death (Xu and Goldkorn, 2016). Interestingly, multiple cancer-specific promoters can be used in combination to further enhance cancer-targeting specificity (Li et al., 2005).

##### Targeting the cancer cell with infectious materials (Figure 4D)

It has been demonstrated that cancer cells are more prone to infection than healthy cells, and that some pathogens tend to favorably infect cancer cells (Marelli et al., 2018; Zhou et al., 2018). As a consequence, strategies targeting cancer cells with infectious materials have been developed. As an example of choice, one of the two approved therapeutic cancer vaccines uses the BCG bacteria as a tumor-killing agent. Currently, most of the infectious-based cancer therapies under development focus on the use of oncolytic viruses, which naturally infect, replicate inside and lyse cancer cells, thus releasing additional viruses in the tumor. Viruses can be engineered at multiple levels to improve their specificity to cancer cells. First, their capsid or envelope can be modified to enhance tropism to cancer cell surfaces (Büning and Srivastava, 2019). For instance, Münch et al. (2013) have modified the capsid of an adeno-associated virus to specifically target HER2-positive tumors upon systemic delivery. Secondly, the virus genome can be manipulated to incorporate cancer-specific promoters, as described above. Similarly, bacteria have been engineered for enhanced cancer-targeting. For instance, *Salmonella typhimurium* decorated with anti-CEA scFv have efficiently targeted CEA-expressing MC38 colon cancer models (Bereta et al., 2007). In cancer vaccines, the use of pathogens as delivery tools is particularly interesting as they naturally contain multiple MAMPs that act as adjuvants.

#### Targeting Tumor-Associated Stromal Cells

Tumors are not only composed of cancer cells, but also of tumor-associated stromal cells (TASCs), including endothelial cells and fibroblasts. Since stromal cells are less heterogenous populations than cancer cells, their markers are more conserved across cancers and patients, possibly enabling the development of less personalized targeting therapies. To date, most strategies that target TASCs focus on the use of antibodies, peptides and small molecules, although gene therapy remains a feasible option. Since TASCs often support tumor development, progression and dissemination, most therapies aim at blocking or killing them to stop tumor nurturing.

##### Targeting tumor-associated blood endothelial cells (BECs)

Because tumors get nutrients and oxygen from blood vessels, many strategies have been developed to prevent tumor angiogenesis and subsequently starve the tumor. Clinically, tumor angiogenesis has been mostly inhibited by blocking the vascular endothelial growth factor-A (VEGF-A), using either blocking antibodies against it or against its receptor VEGF-R2, or tyrosine kinase inhibitors that block VEGF-R2 downstream signaling (Zirlik and Duyster, 2018). Furthermore, tumoral BECs express some surface proteins that are not commonly present in healthy vasculature, such as VEGF-R3 or endoglin, which can be used to preferentially target them (Laakkonen et al., 2007; Dallas et al., 2008). Importantly, it has been shown that modulating tumor vasculature strongly impacts the outcomes of immunotherapies, since blood vessels directly control immune cell trafficking into tumors. Indeed, some anti-angiogenic therapies have been shown to enhance intratumoral trafficking of lymphocytes in some cancers, notably by increasing the expression of adhesion molecules involved in T cell homing and by increased chemokine expression (Tartour et al., 2011; Wu et al., 2016). Alternatively, several strategies have been explored to normalize tumor vasculature rather than blocking it to relieve hypoxia, which has been shown to impair T cell cytotoxic activities (Huang et al., 2013; Uldry et al., 2017).

##### Targeting tumor-associated lymphatic endothelial cells (LECs)

The presence of lymphatic vessels in tumors has been associated with increased metastasis and overall poor diagnosis. Nevertheless, when combined with immunotherapies, lymphatic vessels can instead promote anti-tumor responses by increasing immune infiltrates into tumors, as it has been demonstrated in mouse melanoma models and positively correlated in human melanoma (Fankhauser et al., 2017). By draining tumor interstitial fluid and debris, lymphatic capillaries of LECs are strongly exposed to tumor antigens, which they scavenge and present on their MHCs to further modulate immune responses (Hirosue et al., 2014; Kimura et al., 2015). Due to their endocytic capability, targeting LECs has been achieved passively by injecting drugs upstream to their draining route, using liposomes for instance (Oussoren et al., 1997), although such passive approaches can side-target other phagocytic cells. In contrast, preferential targeting of LECs has been achieved by using anti-VEGF-R3 antibodies (Saif et al., 2016). More recently, a dual targeting approach using anti-Lyve-1 combined with anti-Podoplanin antibodies coated on magnetic nanoparticles has been engineered, although the specificity of this last approach remains to be demonstrated *in vivo* (Wu et al., 2019). Noteworthily, LECs can not only be targeted in the tumor and lymphatic vessels, but also in the lymph nodes where they strongly interact with immune cells.

##### Targeting tumor-associated fibroblasts (TAFs)

Tumor-associated fibroblasts, which constitute a preponderant population in solid tumors, have been shown to promote cancer progression, mediate tumor immunosuppression and affect T cell infiltration (Shiga et al., 2015; Liu et al., 2019), and have therefore been chosen as targets for direct killing. For example, TAFs have been preferentially targeted using lipid-protamine-DNA nanoparticles displaying aminoethylanisamide (AEAA) ligands at their surface, which can bind to their cognate sigma receptors highly expressed by TAFs (Liu et al., 2019). Another interesting approach for the therapeutic use of TAFs in cancer immunotherapy has been proposed by Müller et al. (2008) who have engineered a bispecific antibody-derived fusion protein that targets on one side the Fibroblast Activation Protein (FAP) receptors on TAFs, and on the other side display the extracellular portion of 4-1BBL, a co-stimulatory molecule that promotes T cell activation. This approach of rendering TAFs immunostimulatory could be highly relevant in the context of therapeutic vaccines, to increase intratumoral immunogenicity and endogenous T cell stimulation.

##### Targeting other stromal cells

Other stromal cells present in the tumor microenvironment could be used as potential targets to localize vaccines in the tumor, for example, stromal stem cells such as MSCs (Poggi et al., 2018). Interestingly, MSCs can actively migrate from the systemic circulation into the tumor microenvironment via chemokine gradient sensing, as seen before in the section “Targeting the Tumor Biochemical Environment.”

#### Targeting Tumor Extracellular Matrix (ECM)

In addition to cell targeting, the tumoral ECM has been exploited for the delivery of anti-cancer or immunomodulatory drugs. During tumorigenesis, the ECM is remodeled and dysregulated, leading to changes in composition as compared to healthy ECMs. Such differences in tumor ECM composition make it amenable to targeting of cancer therapeutics. Targeting the tumoral ECM is particularly relevant for the delivery of cytokines or similar signaling molecules, considering the important physiological roles of the ECM in regulating cytokine spatiotemporal release and activity *in vivo* (Frantz et al., 2010; Martino et al., 2014; Briquez et al., 2016). Various components of the ECM have been targeted within tumors, particularly glycoproteins, fibrous scaffold proteins (e.g., collagen), and glycosaminoglycans.

##### Glycoprotein targeting

Some glycoproteins are differently spliced or overexpressed in tumors; for example, the EDA and EDB domains of fibronectin are present in tumors and wounded tissues, but absent in normal matrices. Both of these domains have served as targets for the delivery of cytokines and small molecules into tumoral ECMs (Kaspar et al., 2005; Hutmacher and Neri, 2019). Particularly, the anti-EDB antibody fused to IL-2 and TNF have shown promising results in enhancing anti-tumor immunity and are currently being tested in clinical trials (Hutmacher and Neri, 2019). Other glycoproteins can be similarly targeted, such as tenascin-C or the G45 domain of laminin-332 that are respectively, absent and degraded in physiological ECMs, but often expressed in tumoral matrices. Interestingly, functionalization of drug-loaded nanoliposomes with tenascin-C-binding peptides or sulfatide – a tenascin-C-binding glycosphingolipid – showed successful tumor targeting and reduction of drug side-effects (Lin et al., 2014; Chen et al., 2016b).

##### Collagen targeting

Collagen is another ECM component that can be targeted, as it is present at higher levels in many types of tumors compared to normal tissue (Provenzano et al., 2008; Zhou et al., 2017). Consequently, engineering drugs for collagen targeting can increase *in situ* retention within the tumor. Indeed, intratumoral delivery of IL-2 and IL-12 fused with lumican, a collagen VI-binding protein, has led to increased sustainability, efficacy and safety of these cytokines (Momin et al., 2019). Additionally, collagen is not naturally exposed to the blood stream in healthy tissues, but is accessible upon increase of vessel permeability during inflammation and in cancer. As such, drugs conjugated to appropriate collagen-binding moieties can accumulate in the tumor microenvironment upon systemic delivery (e.g., intravenous). As an example, the fusion of an anti-EGFR Fab to a collagen-binding peptide exhibited localization to A431 xenografts and enhanced retention time compared to untargeted anti-EGFR Fab when injected intraperitoneally (Liang et al., 2016). In addition, a recent study by Ishihara et al. used a collagen I and III-binding domain derived from von Willebrand factor to target checkpoint blockade antibodies and IL-2 to various tumors, which resulted in tumor growth suppression and lowered drug toxicity (Ishihara et al., 2019).

##### Proteoglycan and glycosaminoglycan (GAG) targeting

Similarly to other ECM components, GAGs are also dysregulated in cancers, and many of them, such as hyaluronan (HA) or chondroitin sulfate (CS) are overexpressed by tumor cells (Toole, 2004; Vallen et al., 2014). Accordingly, GAG-binding peptides have been used to target materials into tumors. For instance, silver nanoparticles functionalized with the IP3 HA-binding peptide have successfully localized into peritoneal tumors (Ikemoto et al., 2017). In addition to being overexpressed, some tumoral GAGs also display differences in sulfation patterns, which can be further exploited to enhance targeting specificity (Vallen et al., 2014). As an example, Salanti et al. targeted a specific sulfated form of CS, called CSA, in melanoma and prostate tumors by using a peptide derived from the *Plasmodium falciparum* VAR2CSA protein (Salanti et al., 2015). Other approaches to target CS have used antibody fragments (van der Steen et al., 2017) or liposomes containing a cationic lipid TRX-20 (Lee et al., 2002). Lastly, other GAGs can be used as tumoral ECM targets, notably heparan sulfates and aggrecans (Raavé et al., 2018).

##### Promiscuous ECM Targeting

Finally, a more versatile approach for cancer ECM targeting is to target multiple ECM components at the same time rather than just a single component at a time. For example, this strategy has been exploited by conjugating materials to the heparin-binding domain (HBD) of placenta growth factor-2 (PlGF-2_123__–__144_), which has been shown to display a super-affinity for multiple ECM components (Martino et al., 2014). In one study, PlGF-2_123__–__144_ successfully allowed the retention of checkpoint blockade antibodies within the tumor environment, improving efficacy and safety of these antibodies (Ishihara et al., 2017). However, such an approach is limited to local delivery as it also targets fibrinogen in the blood. Another multi-targeting strategy used a bispecific peptide (PL1) that binds to both fibronectin EDB and tenascin-C. Lingasamy et al. used PL1 to target iron oxide nanoworms loaded with proapoptotic peptides into glioblastoma and prostate carcinoma tumors (Lingasamy et al., 2019).

### Engineering Immune Cell-Targeting Materials

Immune cells are the main actors to mediate tumor cell killing and establish anti-cancer memory. As such, the main challenge of cancer vaccines is to leverage their potential by targeting and stimulating the correct set of immune cells with the appropriate signals. Immune cells can be targeted at various places including in the tumor (depending on the presence of immune infiltrates), in lymphoid organs or in distant tissues, from where they can migrate to the lymphoid organs. As immune cells are dynamically migrating between the different sites, we here classified targeting strategies per cell type rather than by site. We focus on strategies targeting DCs, T cells, B cells, and NK cells as being the most studied targeted cell types for cancer vaccines. However, all immune cells and mechanisms able to mediate or enhance cytotoxicity and memory could play a significant role in the development of future strategies. In particular, macrophages and neutrophils, capable of ADCP, or the complement system are of emerging interests in cancer immunotherapies (Gul and van Egmond, 2015; Reis et al., 2017). Whether their potential could be harnessed to enhance cancer vaccination, however, remains unclear.

#### Targeting Dendritic Cells (DCs)

Dendritic Cells are considered as the most potent APCs and play a pivotal role in triggering adaptive immune responses, due to their ability to promote T and B cell maturation. Consequently, targeting antigens and adjuvants to DCs is an important strategy pursued in cancer vaccines, and has been primarily explored *ex vivo* and more recently *in vivo*.

##### *Ex vivo* DC vaccines

The development of DC-targeting therapies has been made possible by the technical advances in DC isolation and *in vitro* culture. Modifying DCs *ex vivo* has the unique advantage of avoiding the influence of the tumor immunosuppression, which has been shown to impair DCs functions *in vivo* (Pinzon-Charry et al., 2005). Conventional DCs can be isolated from the patient peripheral blood and targeted *in vitro* with both cancer-associated antigens and adjuvants to stimulate their maturation. Delivery of tumor-associated antigens to DCs has been tested in the form of mRNA transfection (Borch et al., 2016), tumor lysates (Yu et al., 2004), co-culture with tumor cells or even fusion of tumor cells with DCs (Yu et al., 2004). Similarly, many adjuvants have been used to mature the DCs, notably exposure to TLR-3, -4, -7/-8, and -9 ligands and co-stimulatory receptor ligands such as CD40L or cytokines (Saxena and Bhardwaj, 2018). Following *in vitro* antigen loading and maturation, DCs are re-administered to the patient via intradermal, intravenous, intranodal or intralymphatic, or intratumoral injection (Shang et al., 2017). Noteworthily, the currently approved therapeutic cancer vaccine Sipuleucel-T for prostate cancer treatment is an *ex vivo* DC-based vaccine.

##### *In vivo* DC-targeting vaccines

While delivered nanomaterials are taken up by DCs *in vivo*, more specific targeting has been achieved by the use of antibodies against DC-specific receptors, such as anti-DEC205, anti-CLEC9A or anti-DC-SIGN (Bonifaz et al., 2004; Hesse et al., 2013; Tullett et al., 2016). Interestingly, it has been shown that targeting different regions on the DC-SIGN receptor can modulate the internalization pathway and influence the extent of antigen presentation on MHCI by DCs (Tacken et al., 2011). In this study, Tacken et al. additionally highlighted that co-targeting of the antigen and adjuvants at the same time using PLGA nanoparticles enhances DC maturation (Tacken et al., 2011; Zitvogel and Palucka, 2011). In addition to antibodies, DCs have been targeted by materials conjugated to mannose or TLR-ligands, the latter being used as targeting tools in addition to being adjuvants (Thomann et al., 2011; Wilson et al., 2019). Upon *in vivo* targeting, DCs migrate to lymph nodes wherein they can efficiently educate T cells. Interestingly, it has been shown that pre-conditioning the vaccine site with pro-inflammatory cytokines enhances DC migration to lymphoid organs (Mitchell et al., 2015).

#### Targeting T Lymphocytes

T lymphocytes are the main actors in anti-tumor cell-mediated cytotoxicity, and their presence in the tumor correlates with good prognosis (Gooden et al., 2011). Currently, the most widely used T cell-targeting strategy relies on antibodies that bind to T cell surface receptors, such as PD1, CTLA4 or LAG3, and has been developed in the context of checkpoint blockade immunotherapies (Grywalska et al., 2018; Havel et al., 2019). In cancer vaccination, however, T cells have been mainly targeted by materials that mimic the role of APCs, aiming at promoting endogenous cancer-specific T cell responses.

##### DC-derived exosomes

One major challenge of DC vaccine remains the control of DC fates upon delivery, which has fostered the development of cell-free alternative strategies. In particular, DCs secrete exosomes that carry antigen-MHC complexes, both MHCI and MHCII, as well as co-stimulatory receptors, which make them capable of activating T cell responses (Zitvogel et al., 1998). DC-derived exosomes have been tested in clinical trials with encouraging outcomes (Morse et al., 2005; Tian and Li, 2017).

##### Artificial APCs (aAPCs)

Similar to the use of exosomes, synthetic materials have been developed to bypass APCs and directly activate T cells *ex vivo* and *in vivo*. Indeed, aAPCs are composed of a biomaterial (lipid-, polymeric- or inorganic-based) with all three signals required for T-cell activation, including MHC-antigen complex, co-stimulatory molecules and cytokines. Generally the co-stimulatory moieties are the antibodies anti-CD3 or anti-CD28 and the cytokines can be any T cell stimulating cytokines, such as IL-2, IL-7, IL-15, or IL-23 (Steenblock et al., 2011; Eggermont et al., 2014; Wang et al., 2017). Interestingly, it has been demonstrated that microparticles are more suited for the design of aAPCs than smaller sized ones as they provide increased interaction with T cells, due to their lower curvature. Particularly, ellipsoidal nanoworm particles induce higher activation efficacy compared to spherical ones (Mandal et al., 2013; Sunshine et al., 2014). In this context, it has been additionally shown that more sustained release of cytokines elicits a stronger immune response. Lastly, these aAPC platforms have also been used to deliver immunosuppressive blocking antibodies and showed promising results in delayed tumor growth (Eggermont et al., 2014; Zhang et al., 2017a).

#### Targeting B Cells

B cells have the dual role of serving as APCs and mediating humoral immune responses. Although the first role has taken more attention in the development of therapeutic cancer vaccines, new strategies are being explored to induce potent anti-tumor immunity relying on humoral responses.

##### B cells as APCs in cancer vaccines

B cells can be turned into potent APCs upon stimulation of their CD40 receptors, using soluble CD40L recombinant proteins, CD40 agonist antibodies or co-culturing them with CD40L-expressing feeder cells (Wennhold et al., 2019). CD40-activated B cells have been then shown to present antigens on both MHCI and MHCII to trigger CD8^+^ and CD4^+^ T cell responses respectively, upon adoptive transfer in humans (Schultze et al., 1997; Lapointe et al., 2003; Wennhold et al., 2019). As a consequence, B cells are being considered as an alternative source of APCs to DCs in cancer vaccines, as they are easier to isolate in sufficient number from patient peripheral blood, even from cancer patients, and are less sensitive to tumor immunosuppression, among other advantages (von Bergwelt-Baildon et al., 2002; Shimabukuro-Vornhagen et al., 2012). In addition, B cells can be targeted *in vivo* for antigen presentation, notably using anti-CD19 antibody as a targeting tool, as exemplified by Ding et al. (2008).

##### Humoral-based cancer vaccines

Another interesting approach relying on B cells is the vaccination against B-cell cancer epitopes, which generates large amounts of endogenous antibodies able to target the cancer cell surface and induce their death via antibody-mediated cytotoxicity (Kaumaya, 2015). As an example, HER2-Vaxx (developed by Imugene) induces polyclonal antibody responses against a specific epitope of HER2 and is being tested in patients with HER2-positive gastric, esophageal, and breast cancers. In the current vaccine, the HER2 peptide is formulated using the carrier protein CRM197, a non-toxic mutant of diphtheria toxin, combined with an adjuvant, whereas the previous formulation used virosome-based materials. This therapy has successfully passed a Phase I safety trial and is under evaluation in a Phase II trial. In addition to direct cytotoxicity, such a B-cell epitope peptide vaccine could provide immune protection against cancer relapse, via the presence of long-term circulating antibodies and memory B cells.

#### Targeting NK Cells

Although NK cells are not the primary targeted cell type in cancer vaccines, they are generating substantial interest due to their cytotoxic ability as well as their strong cooperation with T and B cells. The role of NK cells in cancer immunotherapy is particularly important as they can detect cancer cells that avoid T cell recognition by downregulating their MHC and can mediate antigen-specific ADCC via recognition of cancer cell membrane-bound antibodies (Collins et al., 2011).

Because NK cells are innate immune cells, they do not have, according to our traditional understanding, the ability to mediate direct antigen-specific recognition and immunologic memory, which are central criteria for vaccines. Nevertheless, new roles for NK cells have been unveiled recently, notably in the context of viral infections, suggesting some features of antigen specificity and long-term memory in mouse and primate NK cells (Paust et al., 2010; Reeves et al., 2015; Pahl et al., 2018). Particularly in mice, it has been shown that NK cells can develop specific memory against antigens from murine cytomegalovirus (MCMV), influenza, and vesicular stomatitis virus (VSV) and human immunodeficient virus-1 (HIV), which can be effectively recalled upon antigen re-encounter (Sun et al., 2009; Paust et al., 2010). Currently, researchers are exploring whether such NK memory cells can be harnessed for vaccination purposes in cancer (Sun and Lanier, 2018; Capuano et al., 2019). For example, a study by Romee et al. (2016) has shown that adoptive transfer of cytokine-induced memory-like NK cells reduce leukemia burden in humans.

### Post-targeting Fate of Materials

Targeting the material to the appropriate cells is not sufficient to ensure the therapeutic effect of a cancer vaccine. Indeed, upon targeting, the drugs – antigens and/or adjuvants – remain to be delivered in the appropriate subcellular compartments (i.e., where their targets or receptors are located), which can be the endosomes, cytoplasm, nucleus or other cell organelles. Interestingly, most cell-surface targeting strategies will result in drug internalization in endosomes, yet via multiple pathways (Elkin et al., 2016; Owens et al., 2016). Particularly, from the endosomes, drugs can travel to late endosomes and lysosomes, escape into the cytosol, be recycled at the cell surface or be transported to other organelles such as in the endoplasmic reticulum (Cullen and Steinberg, 2018). Upon escape in the cytosol, drugs can further be directed to the nucleus or to other specific sites, for example targeting mitochondria (Battogtokh et al., 2018). Importantly, the material itself (e.g., size, shapes, ligands, etc.) can influence which internalization pathway will be favored (Gratton et al., 2008).

As a consequence, the type of drug to deliver should be rationally chosen; for example, antigens can be in the form of protein, peptides, RNA or DNA. Importantly, proteinaceous antigens delivered from the extracellular space can be presented on MHCII but less so on MHCI, which is generally restricted to presentation of intracellular antigens. As a consequence, extracellularly-delivered proteins should undergo endosomal escape and reach the cytoplasm to trigger CD8^+^ T cell responses. Accordingly, materials can be engineered to promote endosomal escape (Selby et al., 2017), for example by being pH-sensitive, redox-sensitive, or osmotic change-sensitive to burst the endosomes (Phillips and Gibson, 2014; Kongkatigumjorn et al., 2018; Rangasamy et al., 2018). In addition, some antigenic peptides can be designed to bind to MHCI from the extracellular space, thus bypassing this challenge (Ilca et al., 2018). Interestingly, some APCs subsets (e.g., CD103^+^ DCs) (Joffre et al., 2012) are known to be naturally capable of mounting extracellular antigens onto MHCI, through a mechanism called antigen cross-presentation, and have therefore raised particular attention in vaccination (Fehres et al., 2014). As an alternative to protein forms, antigens can be delivered as DNA or RNA, which are directly translated into the cytosol. This particular advantage has encouraged the development of DNA vaccines to enhance CTL responses (Tiptiri-Kourpeti et al., 2016).

As to adjuvants, their receptors can be similarly located at the cell surface, in the endosomes or intracellularly. Therefore, materials that aim at co-delivering antigens and adjuvants need to be carefully designed in an integrated way.

## Combination of Therapeutic Cancer Vaccines With Other Cancer Therapies

While potent cancer vaccines will strongly activate the immune system to recognize and fight tumors, the activated immune cells still have to infiltrate the tumor and be locally effective to achieve therapeutic effects. These final steps constitute additional challenges, which could be partly overcome by the combination of the vaccine with other cancer therapies.

First, immune cells need to be in contact with the tumor cells to induce their death. The migration of immune cells into tumors is highly dependent on the tumor structure, and can be further dampened through impaired chemokine expression and downregulation of adhesion molecules on endothelial cells (Lanitis et al., 2015; Harjunpää et al., 2019). Consequently, combining cancer vaccines with therapies that ameliorate intratumoral immune infiltrations, such as those modulating local angiogenesis (Wu et al., 2016; Calcinotto et al., 2012) and lymphangiogenesis (Fankhauser et al., 2017), might be highly beneficial.

Secondly, upon reaching the tumor, the immune cells will face an immunosuppressive environment hampering their cytotoxic activity. Indeed, multiple mechanisms are at play in the tumor to prevent tumor cell killing by immune cells, via myeloid-derived suppressive cells, regulatory T cells or the secretion of immunosuppressive factors (e.g., IL-10, TGFβ, IDO) (Binnewies et al., 2018). Nevertheless, the reduction of tumor immunosuppression can potentially be achieved using immune checkpoint blockade, pro-inflammatory cytokines or IDO inhibitors, to mention a few examples.

Lastly, in the tumor, the immune cells still need to detect their targeted antigens in sufficient amount on cancer cells. Particularly, cancer cells that downregulate expression of the antigens or of MHCs will avoid immune recognition and subsequent killing, leading to cancer relapse. Therefore, diversifying antigen targets and immune-mediated cytotoxic mechanisms is essential to reduce the risk of tumor escape. To do so, cancer vaccines can be combined with other therapies that enhance cancer antigen spreading and local DAMPs release from the tumor, such as chemo-, radio- or other immunotherapies (Wang et al., 2018; Joshi and Durden, 2019).

## Conclusion and Perspectives

In conclusion, cancer vaccines hold the promises of eradicating tumors and preventing relapse by inducing strong antigen-specific immune responses and long-term memory. Nevertheless, despite the extensive efforts invested in their development over the last decades, very few have thus far been approved in the clinic. However, lessons from successes, developments-in-progress and failures have increased our knowledge on the design of cancer vaccines, providing some rules to rationally engineer materials that enhance their therapeutic outcomes. Overall, we here proposed that the development of potent vaccines requires careful considerations of their (1) intrinsic composition, i.e., antigens and adjuvants, (2) formulation with materials, (3) delivery route and subsequent targeting to specific relevant sites and cell types, (4) subcellular targeting of their receptors and downstream biological pathways, and (5) combination with other cancer treatments. Although not discussed in this review, the optimal dosing and delivery regimen of the vaccine would also need to be precisely determined, which includes the number of doses to be administered and the delay between repeated delivery.

Defining all these parameters constitutes a major challenge as their combined effects remain poorly predictable, thus requiring thorough investigations *in vitro* and *in vivo*. Therefore, improvement of cancer models for better translatability would be beneficial to select the most relevant formulations and strategies to move forward in the clinic. In addition, establishing which tumor types/subtypes and subset of patients would be the most responsive to a cancer vaccine remains difficult to date, despite advances in diagnostic tools and immunological tests. In that perspective, clinical data collection, standardization and availability are essential to allow meta-analyses that help researchers and clinicians to draw criteria for the vaccine efficacy. Overall, improving the predictability of vaccines’ outcomes would permit to reduce their cost and the time required for their development.

Finally, the vaccine would also need to be manufactured at scale and to comply with the regulatory authorities. The multitude of available materials and engineering strategies could lead to highly complex formulations, optimized for biological efficacy yet challenging to produce and become approved therapies. Therefore, a good compromise between efficacy and feasibility is essential to accelerate clinical translation of therapeutic cancer vaccines in the near future.

## Author Contributions

All authors have contributed in writing of the manuscript. MS and JH have supervised the work and contributed in writing and corrections.

## Conflict of Interest

AT was employed by the company Agap2 Zürich, Switzerland. The remaining authors declare that the research was conducted in the absence of any commercial or financial relationships that could be construed as a potential conflict of interest.
